# Has COVID-19 led to more sudden cardiac deaths in football?

**DOI:** 10.1007/s00392-024-02569-1

**Published:** 2024-11-25

**Authors:** Ana Ukaj, Tim Meyer, Florian Egger

**Affiliations:** Institute of Sports and Preventive Medicine, Saarland University, Campus Geb. B 8.2, 66123 Saarbrücken, Germany

**Keywords:** Sudden cardiac death, Sudden cardiac arrest, Football, COVID-19 pandemic, Myocarditis, Coronary artery disease

## Abstract

**Introduction:**

It is unclear whether the number of sudden cardiac death (SCD) and survived sudden cardiac arrest (SCA) has increased among football players during the COVID-19 pandemic. This study aims to compare the SCD/SCA burden between the pre-pandemic period and COVID-19 pandemic in football players worldwide.

**Methods:**

The COVID-19 pandemic and an equivalent pre-pandemic period (each lasting 1151 days) were analyzed for SCD/SCA by extracting data from the prospective FIFA (Fédération Internationale de Football Association) Sudden Death Registry. Particular focus was placed on cardiac diseases acquired through the novel coronavirus SARS-CoV-2, such as myocarditis and coronary artery disease (CAD), potentially leading to SCD/SCA.

**Results:**

There were 454 SCD/SCA (survival rate: 24%) and 380 SCD/SCA (survival rate: 27%) during the pre-pandemic period and COVID-19 pandemic, respectively (*p* = 0.27). In the pre-pandemic period, out of 191 confirmed and suspected diagnoses, there were 6 (3%) cases of myocarditis and 69 (36%) cases of CAD and during the pandemic out of 136 confirmed and suspected diagnoses, there was 1 (1%) case of myocarditis and 58 (43%) cases of CAD.

**Conclusion:**

The burden of SCD/SCA, particularly myocarditis and CAD, in football players worldwide seemingly has not been higher during the COVID-19 pandemic than during a comparable period before.

**Graphical abstract:**

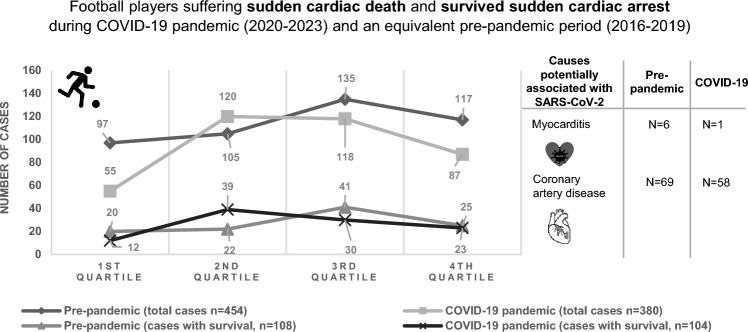

**Supplementary Information:**

The online version contains supplementary material available at 10.1007/s00392-024-02569-1.

## Introduction

The severity of COVID-19 disease is widely determined by co-morbidities and the involvement of extra-pulmonary organs. Since the emergence of COVID-19, cardiac involvement has been documented, such as pericarditis and myocarditis, which may lead to arrhythmia and, in the worst case, to sudden cardiac death (SCD) [1, 2]. There was also emerging evidence that persons suffering from COVID-19 had an increased risk of cardiovascular diseases, among them coronary artery disease (CAD) [3]. This leads to the assumption that the SCD risk in the athlete population may have increased during the pandemic secondary to intense physical activities potentially triggering arrhythmia [4]. Therefore, the main objective of this study is to compare the number of SCD and survived sudden cardiac arrest (SCA) cases in the pre-pandemic period and during the COVID-19 pandemic in football players worldwide using the data from the FIFA Sudden Death Registry [5]. The focus was on cardiac diseases acquired through the novel coronavirus SARS-CoV-2, such as myocarditis and coronary artery disease (CAD), potentially leading to SCD/SCA.

## Methods

This study is an analysis of the numbers and causes of SCD/SCA in the pre-pandemic period and during the COVID-19 pandemic in professional, competitive and recreational football players worldwide. Based on the duration of the COVID-19 pandemic, which lasted 1151 days (11th of March 2020 to 5th of May 2023 according to the World Health Organisation) [6], an equivalent pre-pandemic period (11th of March 2016 until 5th of May 2019) was analyzed and assessed. Data were extracted from the prospective FIFA Sudden Death Registry [5]. This study was approved by the ethics committee and by the independent Data Protection Centers Saarland, Germany (approval number 172/11). Furthermore, for the purpose of this study, the number of cardiac diseases potentially acquired through SARS-CoV-2, such as of CAD and myocarditis, before and during COVID-19 pandemic were extracted. Cases of SCD/SCA were collected from 3 sources [5]:

1. Multilingual freely accessible online platform for reporting cases: https://www.uni-saarland.de/fakultaet-hw/fifa.html.
2. Press monitoring by use of "Meltwater" (Meltwater company, San Francisco, United States of America) covering > 200 countries, 133 languages, and searching 3.1 million articles per day.
3. Exchanging data with collaborating national SCD/SCA registries around the world.

### Statistical analysis

Descriptive statistics were used mainly. Data were expressed as means and standard deviation. Differences in survival rates were analyzed using a two-sided Chi-square test using SPSS (version 18.0). The significance level for the alpha error was set at *p* < 0.05.

## Results

**Fig. 1:**
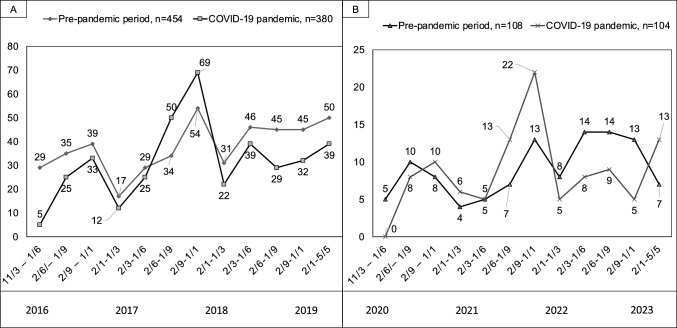
**a** Number of SCD/SCA cases per quarter during the pre-pandemic period and COVID-19 pandemic. **b** Number of SCA cases per quarter during the pre-pandemic period and COVID-19 pandemic.

The demographic data of the football players with SCD/SCA are presented in Table 1. There were 454 SCD/SCA (survival rate: 24%) and 380 SCD/SCA (survival rate: 27%, *p* = 0.27) cases during the pre-pandemic period and COVID-19 pandemic, respectively. In both periods, cases were detected via press monitoring (*n* = 635, 76%), national SCD/SCA registries (*n* = 155, 19%) and the online reporting platform (*n* = 44, 5%). In the pre-pandemic period, from 191 confirmed and suspected diagnoses, there were 6 (3%) cases of myocarditis and 69 (36%) of CAD, and during the pandemic from 136 confirmed and suspected diagnoses, there was 1 (1%) case of myocarditis and 58 (43%) cases of CAD. The most common diagnosis in football players aged > 35 years was CAD before (*n* = 69, 82%) and during the COVID-19 pandemic (*n* = 54, 83%). In football players aged ≤ 35 years, myocarditis was diagnosed in the pre-pandemic period in 4 cases (4%) and during the COVID-19 pandemic in 1 case (1%). The courses of SCD/SCA cases before and during the COVID-19 pandemic are shown in Fig. 1. A list of countries of all reported cases is provided in the supplementary material.

**Table 1:** Demographic data of football players with SCD/SCA during the pre-pandemic period and COVID-19 pandemic

Period	Pre-pandemic period	Pandemic period
Male (*n*, %)	441 (97)	375 (99)
Female (*n*, %)	13 (3)	5 (1)
Number of countries (*n*)	73	73
Recreational level (*n*, %)	196 (43)	115 (30)
Competitive level (*n*, %)	231 (51)	230 (61)
Elite level (*n*, %)	27 (6)	35 (9)
Age (Mean ± SD)*	33 ± 16	31 ± 15

## Discussion

The number of SCD/SCA in football players did not increase during the COVID-19 pandemic when compared to an equivalent period before. Specifically, our data did not show an increase in cases during the COVID-19 pandemic as it would be expected from a higher number of SARS-CoV-2-related myocarditis or CAD. This may be even more significant when considering the raised awareness for CAD and myocarditis during the pandemic as well as the overall upward trend in recorded cases within the FIFA Sudden Death Registry over the years (learning curve) [5].

These numbers cannot explain reasons why there was no increase of SCD/SCA cases, regardless of whether they are due to myocarditis or CAD. One possible explanation is virus-related, i.e., the severity of the infection has not led to relevant additional cardiac damage in this target population [7]. Notably, of 8 athletes with SCA due to myocarditis between January 1, 2020, and June 30, 2022, none were attributed to COVID-19 [8]. Alternatively, management of the disease or its complications might have been improved quickly enough over the pandemic. Additionally, based on large concerns about COVID-19-related myocarditis potentially leading to myocardial injury in young athletes, [9] an extensive screening policy was initiated. This may have led to a relevant proportion of myocarditis cases that were identified and affected players temporarily disqualified from training and competition which may have saved them from SCD/SCA. However, this effect has probably been confined to professional or high-level sport and is, thus, not of major influence on these numbers. Compared to the general population, in which cardiac involvement due to COVID-19 was found in rates up to 4.2%, athletes were shown to have a much lower rate (0.5–2.4%) [10]. In addition, preventive measures such as physical distancing, lockdowns, travel bans and isolation of infected individuals may have limited transmission of SARS-CoV-2 and, thus, the development of myocarditis [11]. Nevertheless, SCD/SCA cases showed a slight upward trend in the summer months during the pandemic (pronounced in the second summer, 2021, Fig. 1), which may be related to fewer contact restrictions. It can be therefore speculated whether the partially enforced training break may have led to a higher risk of resuming football in football players with confirmed cardiovascular involvement due to COVID-19.

### Limitations

It is important to consider the context and limitations of our findings, particularly given the unique circumstances created by the COVID-19 pandemic and the resulting global restrictions on sporting activities. Moreover, the most important limitation of our data is the lack of mandatory case reporting. Reliance on our online reporting platform and media search for data collection may have led to inaccuracies in determining diagnoses. Moreover, the global nature of the FIFA Sudden Death Registry means that data may be subject to different reporting standards and diagnostic criteria in different countries, further impeding interpretation of the results. This could be the reason why the exact cause of death remained unclear in a relevant proportion of cases.

Other reasons for underreporting might be that players took overproportionally long breaks from training after COVID-19 due to concerns about negative consequences or that they were monitored more closely compared to other infections before the pandemic. Moreover, it is possible that an SCD/SCA was not witnessed during individual training due to contact restrictions and therefore not reported in the media, whereas such an event would be very unlikely in an official match.

During the pandemic, especially at its peak, most sporting activities were suspended or severely restricted, with only elite football continuing in many countries. This means that the population studied during the lockdown consisted largely of elite football players, potentially skewing the comparison with a corresponding period before the pandemic, which included a wider range of football playing levels. The restriction to elite players during lockdown could have led to a selection bias, as these athletes typically undergo more rigorous medical screening and monitoring, which could have led to a different baseline risk profile for SCD/SCA. However, the proportion of elite players in our study population was low (< 10% in both periods, Fig. 1) and the potential bias was limited to the cumulative time of national lockdowns, which accounted for a maximum of 24% (9 out of 38 months) of the pandemic period (in most countries < 6 months, 16%) [12].

There was also a lower-than-optimal percentage of autopsy or medical reports due to availability, family interference, country–specific laws, cultural and religious reasons. Still these factors have not changed which makes a biasing influence unlikely. In addition, a lower proportion of myocarditis and CAD cases during the pandemic may to some extent be related to the lockdowns during which football training and matches were suspended. Although, as expected, only a few SCD/SCA were recorded in elite football, it must be taken into account that the potential exposure time was lower due to match cancellations and training interruptions during the COVID-19 pandemic, particularly in amateur football. Nevertheless, adjusting the event rate for the duration of exposure (cases per hour of training or match) was methodologically not possible due to the high variability in lockdown periods in various countries.

Taken together, it is crucial to consider that the data observed during the pandemic may be influenced by several factors, including changes in player health monitoring protocols, the return to high-performance sports after lockdowns and the potential impact of COVID-19 itself. To draw definitive conclusions, further studies are needed that specifically investigate the relationship between vaccination status (difficult to assess) and SCD/SCA in players. The pandemic also led to unprecedented disruptions to training and competition schedules, which could affect players' conditioning and cardiovascular health and potentially increase their susceptibility to SCD/SCA.

## Conclusion

Our data do not support the assumption of an increase in cases of myocarditis, CAD or SCD/SCA in football players during the COVID-19 pandemic compared to pre-pandemic years.

Nevertheless, the potential biases in data collection and the complexity of factors influencing cardiovascular health during this period need to be carefully considered.

## Supplementary Information

Below is the link to the electronic supplementary material.

Supplementary file1 (DOCX 19 KB)

## Data Availability

Data from the registry are not available because identification of individual players may result from that.

## References

[1] Tessitore E, Carballo D, Poncet A, Perrin N, Follonier C, Assouline B, Carballo S, Girardin F, Mach F (2021) Mortality and high risk of major adverse events in patients with COVID-19 and history of cardiovascular disease. Open Heart 8(1):e001526. 10.1136/openhrt-2020-001526 33833064 10.1136/openhrt-2020-001526 PMC8039226

[2] Puntmann VO, Shchendrygina A, Bolanos CR, Madjiguène Ka M, Valbuena S, Rolf A, Escher F, Nagel E (2023) Cardiac involvement due to COVID-19: insights from imaging and histopathology. Eur Cardiol 18(18):e58. 10.15420/ecr.2023.02 37942208 10.15420/ecr.2023.02 PMC10628999

[3] Xie Y, Xu E, Bowe B, Al-Aly Z (2022) Long-term cardiovascular outcomes of COVID-19. Nat Med 28(3):583–590. 10.1038/s41591-022-01689-3 35132265 10.1038/s41591-022-01689-3 PMC8938267

[4] Daniels CJ, Rajpal S, Greenshields JT et al (2021) Prevalence of clinical and subclinical myocarditis in competitive athletes with recent SARS-CoV-2 infection: results from the Big Ten COVID-19 cardiac registry. JAMA Cardiol 6(9):1078–1087. 10.1001/jamacardio.2021.2065 34042947 10.1001/jamacardio.2021.2065 PMC8160916

[5] Egger F, Scharhag J, Kastner A, Dvorak J, Bohm P, Meyer T (2022) FIFA Sudden Death Registry (FIFA-SDR): a prospective, observational study of sudden death in worldwide football from 2014 to 2018. Br J Sports Med 56(2):80–87. 10.1136/bjsports-2020-102368 33361135 10.1136/bjsports-2020-102368

[6] Centers for Disease Control and Prevention. CDC Museum COVID-19 Timeline; March 2023. Available: https://www.cdc.gov/museum/timeline/covid19.html#print.

[7] Moulson N, Petek BJ, Baggish AL, Harmon KG, Kliethermes SA, Patel MR, Churchill TW, Drezner JA (2023) The Cardiac effects of COVID-19 on young competitive athletes: results from the outcomes registry for cardiac conditions in athletes (ORCCA). J Cardiovasc Dev Dis 10(2):72. 10.3390/jcdd10020072 36826568 10.3390/jcdd10020072 PMC9964305

[8] Petek BJ, Churchill TW, Moulson N, Kliethermes SA, Baggish AL, Drezner JA, Patel MR, Ackerman MJ, Kucera KL, Siebert DM, Salerno L, Zigman Suchsland M, Asif IM, Maleszewski JJ, Harmon KG (2024) Sudden cardiac death in National Collegiate Athletic Association athletes: a 20-year study. Circulation 149(2):80–90. 10.1161/CIRCULATIONAHA.123.065908 37955565 10.1161/CIRCULATIONAHA.123.065908 PMC10843024

[9] Drezner JA, Heinz WM, Asif IM, Batten CG, Fields KB, Raukar NP, Valentine VD, Walter KD (2020) Cardiopulmonary considerations for high school student-athletes during the COVID-19 pandemic: NFHS-AMSSM guidance statement. Sports Health. 12(5):459–461. 10.1177/1941738120941490 32640879 10.1177/1941738120941490 PMC7459195

[10] Hofbauer T, Humann K, Neidenbach RC, Scharhag J (2023) Myocarditis screening methods in athletes after SARS-CoV-2 infection—a systematic review. Int J Sports Med 44(13):929–940. 10.1055/a-2099-6725 37225132 10.1055/a-2099-6725

[11] Güner R, Hasanoğlu I, Aktaş F (2020) COVID-19: prevention and control measures in community. Turk J Med Sci. 50(1):571–577. 10.3906/sag-2004-146 32293835 10.3906/sag-2004-146 PMC7195988

[12] The Lost Months of the Coronavirus Pandemic, Oct 21, 2021. https://www.statista.com/chart/26023/areas-with-the-longest-cumulative-pandemic-lockdowns.

