# Supplementary material for: Has COVID-19 led to more sudden cardiac deaths in football?
Source: Clin Res Cardiol. 2024 Nov 25;114(4):492–6. doi: 10.1007/s00392-024-02569-1 (PMC11946988; doi:10.1007/s00392-024-02569-1)
Supplement: Supplementary file 1 — Supplementary file1 (DOCX 19 KB) [file 392_2024_2569_MOESM1_ESM.docx]

**Supplementary material:** Countries with reported SCAs/SCDs in football during the pre-pandemic period and during the COVID-19 pandemic

**Has COVID-19 led to more sudden cardiac deaths in football?**

*Clinical Research in Cardiology*

Ana Ukaj*, Tim Meyer*, Florian Egger

*shared first authorship

^1^ Institute of Sports and Preventive Medicine, Saarland University, Saarbrücken, Germany; FIFA Medical Centre of Excellence

**Corresponding Author:**

Ana Ukaj, MD

PhD candidate

Institute of Sports and Preventive Medicine

Saarland University

Campus Geb. B 8.2

66123 Saarbrücken

Phone: +49 (0) 681 302 70412

[ana.ukaj@uni-saarland.de](mailto:ana.ukaj@uni-saarland.de)

ORCID iDs:

Tim Meyer: 0000-0003-3425-4546

Florian Egger: 0000-0001-5750-2202

|  | **Pre-pandemic period** | **COVID-19 pandemic** |
| --- | --- | --- |
| **1** | Algeria | Algeria |
| **2** | Angola | Argentina |
| **3** | Argentina | Australia |
| **4** | Australia | Austria |
| **5** | Austria | Belgum |
| **6** | Azerbaijan | Benin |
| **7** | Bahrain | Bosnia and Herzegovina |
| **8** | Belgium | Brazil |
| **9** | Bolivia | Cameroon |
| **10** | Bosnia and Herzegovina | Canada |
| **11** | Botswana | Chile |
| **12** | Brazil | China |
| **13** | Cameroon | Colombia |
| **14** | Canada | Croatia |
| **15** | Chile | Denmark |
| **16** | China | Egypt |
| **17** | Colombia | El Salvador |
| **18** | Croatia | Equatorial Guinea |
| **19** | Denmark | Ethiopia |
| **20** | Egypt | France |
| **21** | Eswatini | Gabon |
| **22** | Ethiopia | Germany |
| **23** | France | Greece |
| **24** | French Polynesia | Guatemala |
| **25** | Gabon | Guinea |
| **26** | Georgia | Hungary |
| **27** | Germany | India |
| **28** | Ghana | Indonesia |
| **29** | Greece | Iran |
| **30** | Guatemala | Iraq |
| **31** | Hong Kong | Ireland |
| **32** | Hungary | Israel |
| **33** | India | Italy |
| **34** | Indonesia | Ivory Coast |
| **35** | Ireland | Jamaica |
| **36** | Israel | Kenya |
| **37** | Italy | Kosovo |
| **38** | Ivory Coast | Luxembourg |
| **39** | Jamaica | Malaysia |
| **40** | Kazakhstan | Marocco |
| **41** | Kenya | Mexico |
| **42** | Luxembourg | Moldova |
| **43** | Malawi | Namibia |
| **44** | Malaysia | Netherlands |
| **45** | Mexico | New Zealand |
| **46** | Netherlands | Nigeria |
| **47** | New Zealand | Norway |
| **48** | Nigeria | Oman |
| **49** | Northern Ireland | Pakistan |
| **50** | Paraguay | Panama |
| **51** | Peru | Paraguay |
| **52** | Poland | Philippines |
| **53** | Portugal | Portugal |
| **54** | Romania | Qatar |
| **55** | Russia | Russia |
| **56** | Serbia | Senegal |
| **57** | Sierra Leone | Serbia |
| **58** | Singapore | Slovakia |
| **59** | Slovakia | Slovenia |
| **60** | South Africa | South Africa |
| **61** | Spain | Spain |
| **62** | Sweden | Sri Lanka |
| **63** | Tahiti | Surinam |
| **64** | Tanzania | Togo |
| **65** | Thailand | Turkey |
| **66** | Trinidad-Tobago | Uganda |
| **67** | Turkey | United Arab Emirates |
| **68** | Uganda | United Kingdom |
| **69** | United Kingdom | Uruguay |
| **70** | Uruguay | USA |
| **71** | USA | Venezuela |
| **72** | Vietnam | Zambia |
| **73** | Zimbabwe | Zimbabwe |
